# Assessing the effectiveness of texture and color enhancement imaging versus white‐light endoscopy in detecting gastrointestinal lesions: A systematic review and meta‐analysis

**DOI:** 10.1002/deo2.70128

**Published:** 2025-04-30

**Authors:** Muhammad Shahzil, Talha Bin Kashif, Zainab Jamil, Muhammad Ali Khaqan, Luqman Munir, Zunaira Amjad, Muhammad Saad Faisal, Ammad Javaid Chaudhary, Hassam Ali, Shahryar Khan, Ikponmwosa Enofe

**Affiliations:** ^1^ Department of Internal Medicine Milton S Hershey Medical Center The Pennsylvania State University Hershey Pennsylvania USA; ^2^ Department of Internal Medicine King Edward Medical University, Neela Gumbad Lahore Pakistan; ^3^ Department of Gastroenterology and Hepatology University of Kentucky Lexington Kentucky USA; ^4^ Department of Internal Medicine Services Institute of Medical Sciences Lahore Pakistan; ^5^ Department of Internal Medicine Henry Ford Hospital Detroit Michigan USA; ^6^ Department of Gastroenterology and Hepatology ECU Health Greenville North Carolina USA; ^7^ Department of Internal Medicine The University of Kansas Medical Center Kansas City Kansas USA; ^8^ Department of Gastroenterology and Hepatology Milton S. Hershey Medical Center The Pennsylvania State University Hershey Pennsylvania USA

**Keywords:** diagnostic imaging, early detection of cancer, gastrointestinal endoscopy, gastrointestinal neoplasms, image enhancement

## Abstract

**Introduction:**

Gastrointestinal cancers account for 26% of cancer incidence and 35% of cancer‐related deaths globally. Early detection is crucial but often limited by white light endoscopy (WLE), which misses subtle lesions. Texture and color enhancement imaging (TXI), introduced in 2020, enhances texture, brightness, and color, addressing WLE's limitations. This meta‐analysis evaluates TXI's effectiveness compared to WLE in gastrointestinal lesion lesion detection.

**Methods:**

A systematic review and meta‐analysis were conducted per Preferred Reporting Items for Systematic Reviews and Meta‐Analyses guidelines. Searches of CENTRAL, PubMed, Embase, and Web of Science identified randomized controlled trials and observational studies comparing TXI with WLE. Outcomes included lesion detection rates, color differentiation, and visibility scores. The risk of bias was assessed using the Cochrane ROB 2.0 tool and Newcastle‐Ottawa tools, and evidence certainty was evaluated using Grading of Recommendations Assessment, Development, and Evaluation.

**Results:**

Seventeen studies with 16,634 participants were included. TXI significantly improved color differentiation (mean difference: 3.31, 95% confidence interval [CI]: 2.49–4.13), visibility scores (mean difference: 0.50, 95% CI: 0.36–0.64), and lesion detection rates (odds ratio [OR]: 1.84, 95% CI: 1.52–2.22) compared to WLE. Subgroup analyses confirmed TXI's advantages across pharyngeal, esophageal, gastric, and colorectal lesions. TXI also enhanced adenoma detection rates (OR: 1.66, 95% CI: 1.31–2.12) and mean adenoma detection per procedure (mean difference: 0.48, 95% CI: 0.25–0.70).

**Conclusion:**

TXI improves gastriontestinal lesion lesion detection by enhancing visualization and color differentiation, addressing key limitations of WLE. These findings support its integration into routine endoscopy, with further research needed to compare TXI with other modalities and explore its potential in real‐time lesion detection.

## INTRODUCTION

Gastrointestinal cancers are one of the most prevalent cancers globally, with over 4.8 million new cases and 3.4 million deaths annually.^1^ They account for approximately 26% of the global cancer incidence and 35% of all cancer‐related fatalities.^1^ An important concern for GI malignancies is diagnosis at an advanced stage, resulting in higher morbidity; however, this can often be avoided if detected and resected at an earlier stage.^2^ Endoscopy is the most frequently employed method for diagnosing GI diseases including malignancies. White light endoscopy (WLE) has been conventionally used to detect mucosal puffiness, ulcerative masses, and stem polyps. However, it falls short in identifying small polyps and flat lesions, often missing many of these abnormalities.^3^ Research indicates that standard white light imaging (WLI) during endoscopy has low detection rates, missing 25% of colonic lesions, 4.6% to 25.8% of gastric lesions, 45% of esophageal squamous cell carcinoma (ESCC), and 92% of superficial pharyngeal squamous cell carcinoma (SPSCC).^4^, ^5^ These limitations prompted the development of new image‐enhanced endoscopy (IEE) modalities such as flexible spectral imaging, narrow‐band imaging (NBI), linked color imaging (LCI), blue laser imaging, and texture and color enhancement imaging (TXI). IEE has recently garnered significant attention for its potential application in diagnosing a variety of gastroenterological lesions (including small and flat lesions) earlier than WL.^6^

TXI was introduced as a new optical IEE modality in April 2020 by Olympus Corporation in Tokyo, Japan^7^ It uses Retinex theory‐based image processing technology to enhance three imaging factors in WLI; thst is, texture, brightness, and color.^8^, ^9^ Retinex is founded on the theory of “color constancy” and “brightness constancy” which pertain to how the human eye perceives color and brightness consistently, regardless of the lighting conditions.^10^ TXI, thus, generally outperforms WLI in detecting and visualizing lesions in GI diseases, including pharyngeal and ESCC, gastric cancer, gastric atrophy, and intestinal metaplasia,^8^ as it is personalized for the human eye and selectively enhances brightness in darker areas of an endoscopic image and highlights subtle tissue differences, including slight morphological or color changes while avoiding over‐enhancement. Studies highlight the potential of TXI as a future clinical tool for detecting GI lesions with subtle tissue differences that are challenging to identify.^11^

The current randomized controlled trials (RCTs) comparing TXI and WLE contain relatively small study populations therefore, a systematic compilation of all available RCTs would boost statistical power and potentially strengthen the confidence in the findings. This review seeks to educate and guide practicing physicians, while also laying a foundation for future researchers to expand the scientific understanding of this modality. To the best of our knowledge, this is the first meta‐analysis encompassing all the available evidence on the effectiveness of TXI compared to WLE.

## MATERIALS AND METHODS

This meta‐analysis was conducted in accordance with the guidelines outlined in the Cochrane Handbook for Systematic Reviews of Interventions and is reported following the Preferred Reporting Items for Systematic Reviews and Meta‐Analyses (PRISMA) statement.^12^ The study was prospectively registered with PROSPERO (CRD42025639550). Since the study synthesized data from existing literature, ethical approval was not required.

### Information sources

A comprehensive literature search was performed across multiple databases, including the Cochrane Central Register of Controlled Trials (CENTRAL), PubMed, Embase (Elsevier), and Web of Science. The search covered the period from each database's inception up to April 2024 to capture all relevant studies. Both RCTs and observational studies—including prospective and retrospective cohort studies and case‐control studies—were considered. The search utilized a combination of Medical Subject Headings (MeSH) and free‐text terms, including “Texture and Color Enhancement,” “TXI,” “White Light Endoscopy,” and “WLE.” A detailed search strategy, including specific search strings, is provided in File S1 (Table S1).

### Eligibility criteria

Studies were included if they met the following criteria: they were RCTs or observational studies (prospective or retrospective cohort studies, case‐control studies); they involved adult patients aged 18 years or older undergoing assessment of GI lesions—including pharyngeal, esophageal, stomach, small intestine, and colon lesions; compared patients undergoing TXI endoscopy (intervention group) with those undergoing WLE endoscopy (comparator group); and reported at least one of the following outcomes—color difference (CD) between lesion and surrounding mucosa, visibility score of the lesion, or GI lesion detection rate.

Studies were excluded if they were case reports, case series, single‐arm studies, guidelines, duplicate publications, conference proceedings, animal studies, unpublished non‐peer‐reviewed articles, or review articles. In instances where multiple studies reported overlapping data, the most comprehensive and recent publication was included to avoid duplication.

### Selection process

**FIGURE 1:**
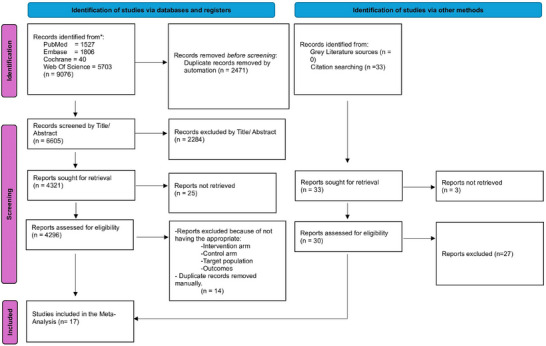
Preferred Reporting Items for Systematic Reviews and Meta‐Analyses (PRISMA) flow diagram. Study selection process following PRISMA guidelines. The flow diagram outlines identification, screening, eligibility, and inclusion stages, with reasons for exclusion at various stages.

All identified articles were imported into Mendeley (version 1.19.8) for the removal of duplicates. Two independent reviewers (Muhammad Shahzil and Talha Bin Kashif) screened the titles and abstracts for relevance. Full‐text articles of potentially eligible studies were then assessed against the inclusion criteria. Any discrepancies between the reviewers were resolved through discussion or, if necessary, consultation with a third reviewer (Zainab Jamil). The study selection process is illustrated in a PRISMA flowchart (Figure 1).

### Data collection process

Data extraction was conducted independently by two reviewers (Muhammad Shahzil and Talha Bin Kashif) using a predefined Excel template. Extracted information included study characteristics (author, year of publication, country, and study design), participant characteristics (age, gender, and lesion size), details of the interventions and comparators, outcomes measured, and duration of follow‐up. Any disagreements were resolved by consensus or by involving a third reviewer (Zainab Jamil) to ensure the accuracy and completeness of the data.

### Outcomes

The primary outcomes were the CD between the lesion and surrounding mucosa, the visibility score of the lesion, and the GI lesion detection rate. Secondary outcomes included visibility scores stratified by lesion characteristics (vessel pattern, surface pattern, margin pattern), visibility scores for sessile serrated lesions and hyperplastic polyps (colorectal lesions), mean adenoma detection rate per procedure (colorectal lesions), and colorectal polypoid adenoma detection rate.

A subjective visibility scoring system was used, with the visibility scale defined as follows: 1 for poor (not detectable without repeated careful examination), 2 for fair (hardly detectable without careful examination), 3 for good (detectable with careful observation), and 4 for excellent (easily detectable). For color analysis, endoscopic images were evaluated and scored based on L* a* b* values, where L* represents light/dark, a* represents red/green, and b* represents yellow/blue, within the Commission Internationale de l'Eclairage (CIE) L* a* b* color‐space system.^29^

### Risk of bias and quality of evidence assessment

The quality of the included studies was assessed using the Cochrane Risk of Bias 2.0 tool for randomized trials and the Newcastle‐Ottawa Scale (NOS) for observational studies.^13^ Each study was independently evaluated by two reviewers (MS and TK) across relevant domains such as selection bias, performance bias, detection bias, attrition bias, and reporting bias. Judgments were categorized as low risk, some concerns, or high risk of bias. The Grading of Recommendations Assessment, Development, and Evaluation (GRADE) approach was employed to assess the certainty of evidence for each outcome, considering factors like study limitations, consistency of results, precision, directness, and potential publication bias.^14^ Detailed assessments are provided in File S1 (Table S2).

### Statistical analysis

Meta‐analyses were performed using RevMan Web (The Cochrane Collaboration, Copenhagen, Denmark), employing a random‐effects model to account for variability among studies. Dichotomous outcomes were expressed as Odds ratios (OR) with 95% confidence intervals (CIs), while continuous outcomes were presented as mean differences (MD) with 95% CIs. A p‐value of less than 0.05 was considered statistically significant.

Heterogeneity among studies was assessed using the Chi‐square test (with significance set at *p*  <  0.10) and quantified using the Higgins I^2^ statistic. I^2^ values of 25%, 50%, and 75% were considered to represent low, moderate, and high heterogeneity, respectively. In cases where substantial heterogeneity was detected (I^2^  >  50%), potential sources were explored through subgroup analyses and sensitivity analyses.

Publication bias was evaluated using funnel plots for outcomes reported in more than three studies (File S2). Sensitivity analyses were conducted by excluding studies identified as having a high risk of bias to assess the robustness of the findings. All statistical analyses adhered to the guidelines provided in the Cochrane Handbook for Systematic Reviews of Interventions and the methodological recommendations by Rücker et al.^15^ Continuous and dichotomous data were combined following the appropriate formulas and guidance to ensure accurate synthesis of the evidence.

## RESULTS

### Study selection and baseline characteristics

Following PRISMA guidelines, our systematic review and meta‐analysis incorporated 17 studies, consisting of three RCTs,^5^, ^16^, ^17^ eight retrospective observational studies,^7^, ^18^, ^19^, ^20^, ^21^, ^22^, ^23^, ^24^ and six prospective observational studies.^4^, ^6^, ^10^, ^25^, ^26^, ^27^ Notably, one study was a multicenter international trial, another was conducted in Australia, while the remaining studies were conducted in Japan. Participant ages ranged from 55 to 75 years, with a total sample size of 16,634. The TXI group comprised 4,293 participants, whereas the WLE group included 12,341 participants. Detailed demographic and baseline characteristics are provided in Table 1a and Table 1b.

**TABLE 1a:** Baseline characteristics of included studies: baseline characteristics of the included studies, detailing study design, intervention, and control groups, total participants, number of lesions, and target lesion types. Texture and color enhancement imaging (TXI) refers to texture and color enhancement imaging, and white light endoscopy (WLE) refers to white‐light imaging.

						*Total participants (n/N)*			
*Study*	*Year*	*Country*	*Study design*	*Intervention (TXI)*	*Control (WLI)*	*Intervention (TXI)*	*Control (WLI)*	*Number of lesions (n)*	*Target lesion*
***Antonelli***	**2023**	Germany, Italy, and Japan	Multicenter Randomized Controlled Trial	TXI	WLI Endoscopy	375	372	–	Colorectal adenomas
***Abe***	**2021**	Japan	Post hoc analysis	TXI‐1, TXI‐2	WLI Endoscopy	18	18	20	Early gastric cancer (EGC), gastric adenocarcinoma
***Okumura***	**2024**	Japan	Retrospective, single‐center study	TXI‐1	WLI, NBI	42	42	100	Non‐polypoid colorectal lesions
***Kato***	**2024**	Japan	Retrospective study	TXI‐1	WLI, NBI	20	20	20	Esophageal squamous cell carcinoma
***Kemmoto***	**2023**	Japan	Retrospective, single‐center observational study	TXI‐2	WLI	2695	10745	50	Gastric cancer
***Dobashi***	**2021**	Tokyo, Japan	Prospective single‐arm observational study	TXI‐1, TXI‐2	WLI in the same arm	‐	‐	59	Pharyngeal and esophageal
***Sugimoto***	**2022**	Tokyo, Japan	Retrospective single‐arm observational study	TXI	WLI in the same arm	40	40	–	Barrett's esophagus and reflux esophagitis
***Sakamoto***	**2023**	Japan	Retrospective single‐arm observational study	TXI	WLI	470	470	1043	Colorectal adenomas
***Ikeda***	**2023**	Tokyo, Japan	Prospective single‐arm study	TXI‐1, TXI‐2	WLI	52	52	52	Barrett's esophagus
***Young***	**2023**	South Australia	Multicenter randomized controlled trial	TXI‐1	WLI	163	161	–	Colonic adenomas
***Yoshida***	**2021**	Japan	Retrospective single‐arm study	TXI‐1	WLI in the same arm	26	26	101	Colorectal non‐polypoid lesions
***Futakuchi***	**2023**	Tokyo, Japan	Prospective single‐arm study	TXI‐1, TXI‐2	WLI in the same arm	49	49	52	Gastric neoplasms
***Ishikawa***	**2021**	Japan	Prospective single‐arm observational study	TXI‐1, TXI‐2	WLI in the same arm	19	19	12 and 20	Gastric mucosal atrophy and gastric neoplasms
***Koyama***	**2023**	Tokyo, Japan	Retrospective single‐arm observational study	TXI‐2	WLI in the same arm	31	31	31	Gastric neoplasms
***Nishizawa***	**2021**	Japan	Prospective single‐arm observational study	TXI‐1, NBI, Chromoendoscopy	WLI in the same arm	27	27	29	Serrated colorectal lesions
***Toyoshima***	**2022**	Tokyo, Japan	Prospective single‐arm observational study	TXI‐1, NBI, Chromoendoscopy	WLI in the same arm	37	37	61	Colorectal adenomas
***Hiramatsu***	2023	Japan	Retrospective single‐arm observational study	TXI‐1, CE, TXI+CE (chromoendoscopy)	WLI	48	48	81	Colorectal polyps

Abbreviations: CE, Chromoendoscopy; CRC, Colorectal cancer.; GC, Gastric cancer; NBI, Narrow‐band imaging; RCT, Randomized controlled trial; TXI, Texture and color enhancement imaging; WLI, White‐light imaging.

**TABLE 1b:** Participant demographics and clinical information: demographic and clinical details of participants, including mean age, sex distribution, *H. pylori* status, operator skill level, and indications for intervention. Data are stratified by intervention (TXI) and control (WLE) groups.

	*Mean age (± SD)*		*Male participants (n/N)*		*Female participants (n/N)*		*H. pylori status (n/N)*			
*Study*	*Intervention (TXI)*	*Control (WLI)*	*Intervention (TXI)*	*Control (WLI)*	*Intervention (TXI)*	*Control (WLI)*	*Intervention (TXI)*	*Control (WLI)*	*Operator skill level*	*Indication for intervention*
***Antonelli***	62.8 (9.6)	62.2 (9.3)	187/375	184/372	187/375	188/372	–	–	Experienced endoscopists (> 2000 screening colonoscopies)	Primary CRC screening, post‐polypectomy surveillance, follow‐up for positive fecal immunochemical test, or other symptoms/signs
***Abe***	64.67 (35.40)	64.67 (35.40)	13/18	13/18	45,430	45,430	Positive: 2/18 Negative: 4/18 Eradicated: 12/18	Positive: 2/18 Negative: 4/18 Eradicated: 12/18	Two expert endoscopists recognized by the Japanese Gastroenterological Endoscopy Society	Cases of early gastric cancer (EGC) clinically diagnosed as gastric adenocarcinoma, meeting the criteria for endoscopic submucosal dissection (ESD) in Japanese Guidelines.
***Okumura***	65.9 (10.5)	65.9 (10.5)	34/42	34/42	15,554	15,554	–	–	Ten endoscopists: five novices (< 500 colonoscopies) and five experts (> 5000 endoscopies with NBI/TBI experience)	Diagnosed cases of non‐polypoid colorectal lesions
***Kato***	70.33 (15.96)	70.33 (15.96)	18/20	18/90	45,332	45,332	–	–	Five expert endoscopists board‐certified by the Japan Gastroenterological Endoscopy Society	Patients with esophageal squamous cell carcinoma (ESCC) who underwent esophageal submucosal dissection
***Kemmoto***	58 (51.16)	58 (51.16)	1651/2695	6495/10,745	1044/2695	4250/10,745	Positive: 108/2695 Negative: 1556/2695 Eradicated: 1031/2695	Positive: 399/10,745 Negative: 6123/10,745 Eradicated: 4223/10,745	Twenty endoscopists; WLI‐expert subset includes top three endoscopists with high gastric cancer (GC) detection rates	Patients with confirmed Helicobacter pylori infection undergoing screening endoscopy
***Dobashi***	–	–	–	–	–	–	–	–	Single expert endoscopist	Patients with superficial neoplasms, including squamous cell carcinoma (SCC) or intraepithelial neoplasia (IN) of the pharynx or esophagus
***Sugimoto***	74.2 (5.8)	74.2 (5.8)	25/40	25/40	15/40	15/40	Positive: 0/40, Negative: 3/40, Eradicated: 37/40	Positive: 0/40, Negative: 3/40, Eradicated: 37/40	Expert endoscopists	Patients with Barrett's esophagus and reflux esophagitis
***Sakamoto***	64.2 (12.1)	63.7 (12.8)	154/237	147/233	83/237	86/233	–	–	Thirty‐five endoscopists	CRC screening (positive fecal occult blood test), post‐treatment surveillance, workup for lower GI symptoms, polyp follow‐up, and pretreatment workup
***Ikeda***	64.1 (14.2)	64.1 (14.2)	25/52	25/52	27/52	27/52	Positive: 5/52 Eradicated: 24/52	Positive: 5/52 Eradicated: 24/52	Ten endoscopists (five experts, five trainees)	Gastroesophageal reflux disease (GERD) symptoms, medical check‐ups, anemia, abdominal pain, follow‐up after gastric ulcers
***Young***	59.67 (14.21)	61 (13.46)	87/163	78/161	76/163	83/161	–	–	Four proceduralists with at least five years of experience	–
***Yoshida***	66.5 (10.3)	66.5 (10.3)	19/26	19/26	45499	45,499	–	–	Three expert endoscopists	–
***Futakuchi***	63.83 (40.48)	63.83 (40.48)	37/49	37/49	13,850	13,850	Positive: 16/49 Negative: 12/49 Eradicated: 24/49	Positive: 16/49 Negative: 12/49 Eradicated: 24/49	Six endoscopists (three experts with > 500 cases and three novices)	–
***Ishikawa***	73.0 (9.0)	73.0 (9.0)	45,645	45,645	45,492	45,492	19/19	19/19	Two experts	–
***Koyama***	73.2 (8.7)	73.2 (8.7)	24/31	24/31	45,504	45,504	Positive: 7/31 Negative: 2/31 Eradicated: 21/31 Undetermined: 1	Positive: 7/31 Negative: 2 Eradicated: 21/31 Undetermined: 1	Ten endoscopists (five experts, five trainees)	–
***Nishizawa***	–	–	–	–	–	–	–	–	Three expert endoscopists	–
***Toyoshima***	59.1(9.0)	59.1(9.0)	19/37	19/37	18/37	18/37	–	–	–	Screening, examination for symptoms, investigation following positive fecal immunohistochemical test, and polyp surveillance
***Hiramatsu***	–	–	–	–	–	–	–	–	Three endoscopists	–

Abbreviations: EGC, Early gastric cancer; ESCC, Esophageal squamous cell carcinoma; GERD, Gastroesophageal disease; GI, Gastrointestinal.; H. pylori, Helicobacter pylori; TXI, Texture and color enhancement imaging; WLI, White‐light imaging.

### Quality assessment and publication bias

**FIGURE 2:**
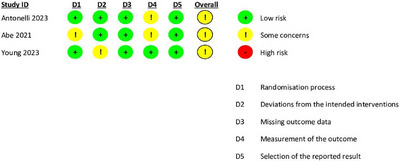
Risk of bias (ROB) assessment. Risk of bias assessment of included studies categorized into key domains, summarizing the overall methodological quality of the studies included in the analysis.

We assessed the risk of bias using the RoB 2.0 tool for randomized studies and the NOS for observational studies. Most RCTs demonstrated moderate quality, as shown in (Figure 2). Among the observational studies, all received a “good quality” rating, scoring seven or above on the NOS in alignment with the Agency for Healthcare Research and Quality standards (Table 2).

**TABLE 2:** Newcastle‐Ottawa Scale (NOS) quality assessment of included studies: quality assessment scores for included studies using the Newcastle‐Ottawa Scale. The scores are presented in three domains: selection, comparability, and outcome, with a cumulative score reflecting the overall study quality.

Author	Selection	Comparability	Outcome	Cumulative Score
Kato, 2024	2	2	3	7
Kemmoto, 2023	3	1	2	6
Dobashi, 2021	4	1	2	7
Sugimoto, 2022	3	2	1	6
Ikeda, 2023	3	2	3	8
Yoshida, 2021	3	3	1	7
Futakuchi, 2023	3	4	2	9
Ishikawa, 2021	2	2	1	5
Koyama, 2023	4	1	3	8
Nishizawa, 2021	3	2	3	8
Toyoshima, 2022	3	3	2	8
Hiramatsu, 2023	3	1	3	7
**Yoshida, 2022**	4	2	3	9
**Sakamoto, 2022**	4	2	3	9

**Abbreviations**: NOS, Newcastle‐Ottawa Scale; TXI, Texture and color enhancement imaging; WLI, White‐light imaging.

## PRIMARY OUTCOMES

### Color difference between the lesion and surrounding mucosa

#### Overall results

**FIGURE 3:**
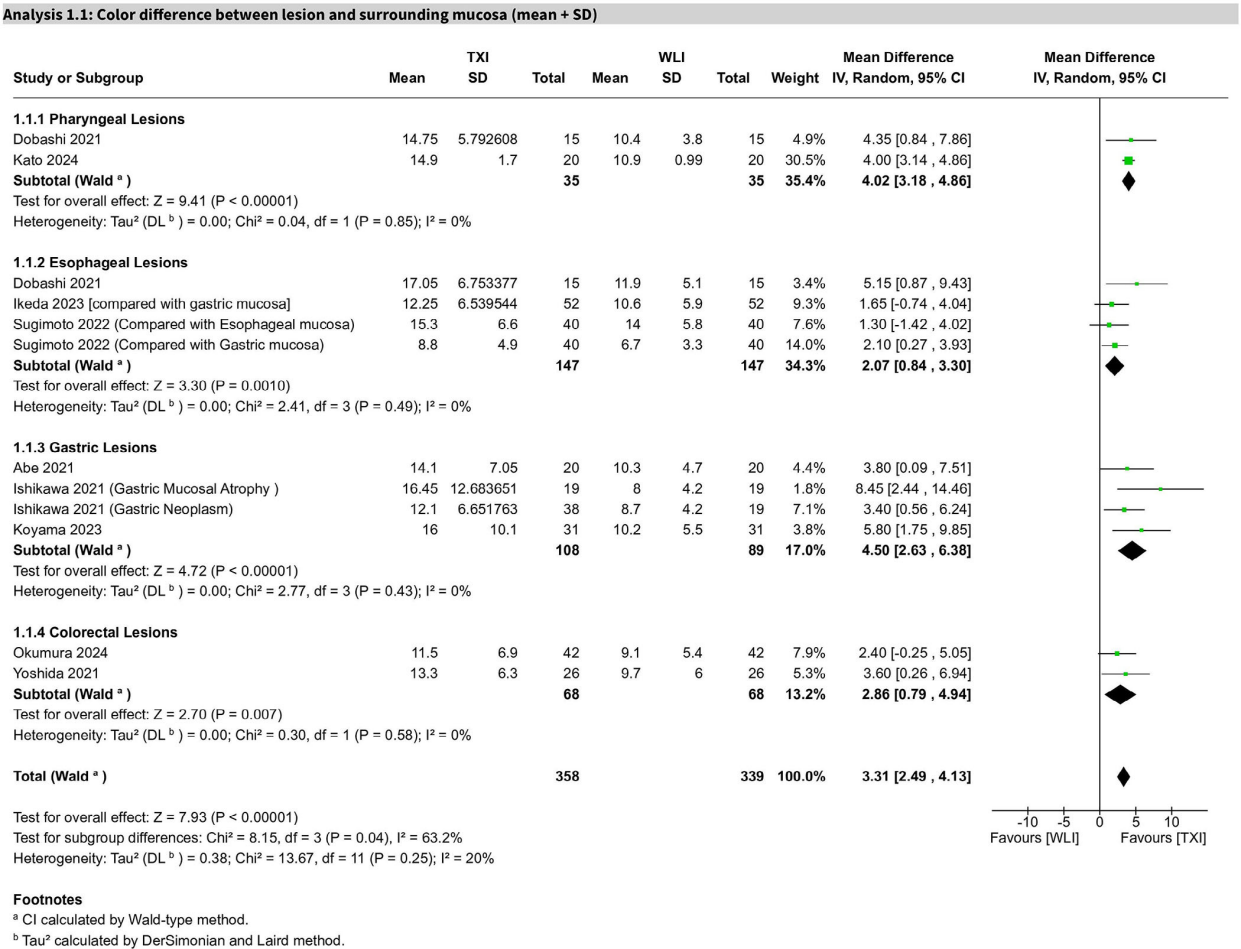
Forest plot for primary outcome: color difference. Forest plot showing the pooled analysis of the color difference between lesions and surrounding mucosa for texture and color enhancement imaging (TXI) versus white light endoscopy (WLE). The plot presents mean differences, 95% confidence intervals, and heterogeneity statistics.

Based on 11 studies, TXI significantly improved the CD between GI lesions and surrounding mucosa compared to WLE (MD 3.31; 95% CI: 2.49–4.13; I^2^ = 20%). This effect was statistically significant, with high certainty of evidence per GRADE (Figure 3).

#### Subgroup analysis

In pharyngeal lesions (two studies), TXI showed a significant improvement (MD 4.02; 95% CI: 3.18–4.86; I^2^ = 0%). In esophageal lesions (five studies), the effect was also significant (MD 2.93; 95% CI: 1.24–4.62), with moderate heterogeneity (I^2^ = 55%). Sensitivity analysis excluding one study reduced heterogeneity (I^2^ = 0%) and yielded a significant MD of 2.07 (95% CI: 0.84–3.30; Figures S1 and S2). For gastric lesions (4 studies), the result was significant (MD 4.50; 95% CI: 2.63–6.38; I^2^ = 0%), as well as for colorectal lesions (2 studies; MD 2.86; 95% CI: 0.79–4.94; I^2^ = 0%).

### Visibility score of the lesion

#### Overall results

**FIGURE 4:**
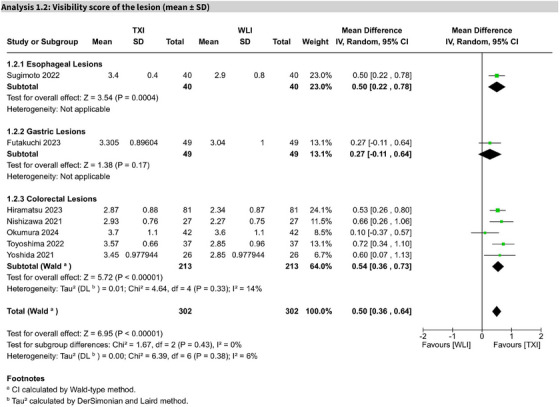
Forest plot for visibility scores by lesion type. Pooled analysis of visibility scores stratified by lesion type, including colorectal, gastric, and esophageal lesions. Forest plots display mean differences, confidence intervals, and heterogeneity measures for texture and color enhancement imaging (TXI) versus white light endoscopy (WLE).

Seven studies showed that TXI significantly improved lesion visibility scores compared to WLE (MD 0.50; 95% CI: 0.36–0.64; I^2^ = 6%). GRADE‐rated certainty was high (Figure 4).

#### Subgroup analysis

For esophageal lesions, one study on Barrett's esophagus showed significant improvement (MD 0.50; 95% CI: 0.22–0.78). One study on gastric neoplasms (including adenoma and adenocarcinoma) reported a non‐significant difference (MD 0.27; 95% CI: −0.11 to 0.64). For colorectal lesions (five studies), visibility scores significantly improved with TXI (MD 0.54; 95% CI: 0.36–0.73; I^2^ = 14%).

### Gastrointestinal lesion detection rate

#### Overall results

**FIGURE 5:**
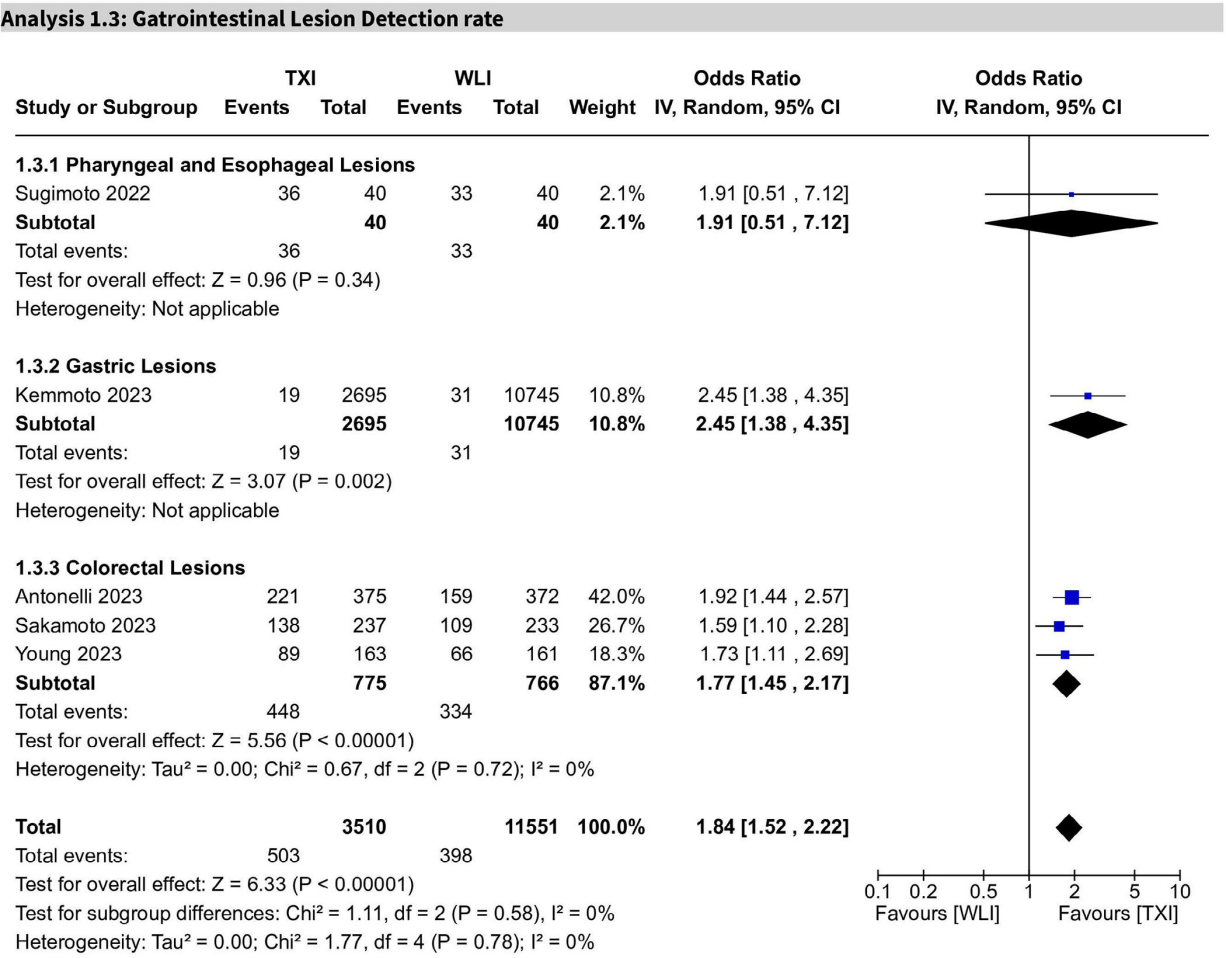
Forest plot for gastrointestinal lesion detection rate by lesion location. Forest plot showing the gastrointestinal lesion detection rates stratified by lesion location: pharyngeal and esophageal lesions, gastric lesions, and colorectal lesions. Odds ratios and 95% confidence intervals are presented for each subgroup, with significant advantages for texture and color enhancement imaging (TXI) observed in gastric and colorectal lesions.

Across five studies, TXI significantly increased GI lesion detection rates (OR 1.84; 95% CI: 1.52–2.22; I^2^ = 0%). GRADE‐rated certainty of evidence was moderate (Figure 5).

#### Subgroup analysis

In pharyngeal and esophageal lesions (one study), the result was not statistically significant (OR 1.91; 95% CI: 0.51–7.12). For gastric lesions (1 study), TXI showed a significant improvement (OR 2.45; 95% CI: 1.38–4.35; I^2^ = 0%). In colorectal lesions (3 studies), detection was significantly enhanced (OR 1.77; 95% CI: 1.45–2.17; I^2^ = 0%).

### TXI‐mode 1 versus TXI‐mode 2

**FIGURE 6:**
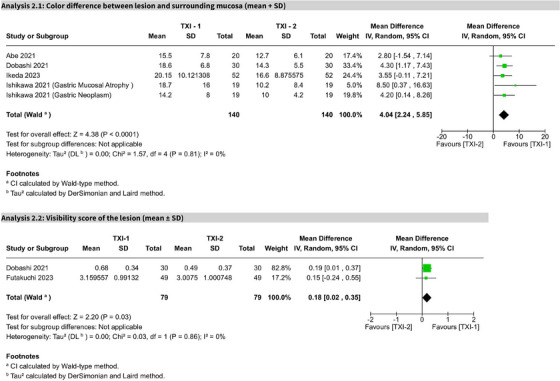
Forest plot: comparison of texture and color enhancement imaging (TXI)‐mode 1 vs TXI‐mode 2. Forest plot presenting pooled mean differences comparing TXI‐Mode 1 and TXI‐Mode 2 for color differentiation and lesion visibility scores, highlighting the superiority of TXI‐Mode 1.

In five studies, TXI‐Mode 1 significantly outperformed Mode 2 in CD (MD 4.04; 95% CI: 2.24–5.85; I^2^ = 0%). Two studies assessing visibility scores also favored TXI‐1 (MD 0.18; 95% CI: 0.02–0.35; I^2^ = 0%). Both outcomes were statistically significant and rated as high certainty per GRADE (Figure 6).

### Experts versus trainees

**FIGURE 7:**
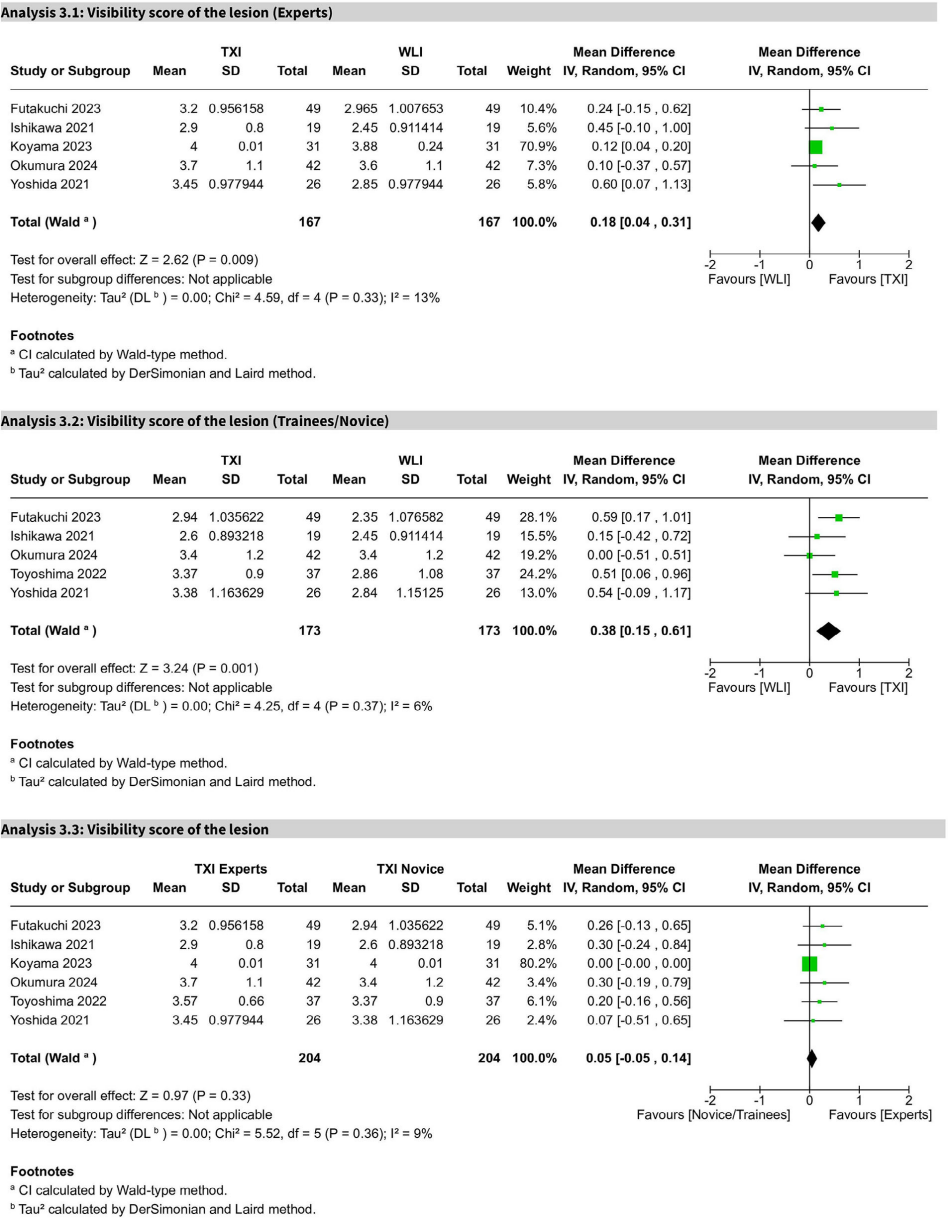
Forest plot: experts versus trainees in lesion visibility. Forest plot comparing lesion visibility scores between expert endoscopists and trainees/novices using texture and color enhancement imaging (TXI), demonstrating similar effectiveness across different levels of endoscopic experience.

Five studies showed TXI significantly improved visibility scores for experts (MD 0.18; 95% CI: 0.04–0.31; I^2^ = 13%) and trainees (MD 0.38; 95% CI: 0.15–0.61; I^2^ = 6%) compared to WLE. A direct comparison within the TXI group showed no significant difference between experts and trainees (MD 0.05; 95% CI: −0.05 to 0.14; I^2^ = 9%). All comparisons were of high certainty (Figure 7 and Figures S3–S6).

## SECONDARY OUTCOMES

### Visibility score stratified by lesion characteristics

For vessel pattern visibility (two studies), TXI significantly improved scores (MD 0.75; 95% CI: 0.47–1.03; I^2^ = 0%). Surface pattern visibility (three studies) showed a significant effect (MD 0.82; 95% CI: 0.60–1.04; I^2^ = 0%). Margin pattern visibility (2 studies) also improved (MD 0.55; 95% CI: 0.23–0.87; I^2^ = 0%). All findings were statistically significant but rated as low certainty (GRADE, Figure S7). The visibility of sessile serrated lesions and hyperplastic polyps (three studies) significantly improved with TXI (MD 0.57; 95% CI: 0.14–0.99; I^2^ = 50%), with GRADE‐rated certainty as low (Figure S8).

### Colorectal adenoma detection

Two studies reported a significant improvement in adenoma detection with TXI (OR 1.66; 95% CI: 1.31–2.12; I^2^ = 0%). The certainty of the evidence was moderate. The mean adenoma detection rate per procedure (three studies) was also significantly higher with TXI (MD 0.48; 95% CI: 0.25–0.70; I^2^ = 0%). The certainty of evidence was moderate (Figure S8).

## DISCUSSION

Our comprehensive meta‐analysis of RCTs is the first to demonstrate a statistically significant advantage of TXI over WLE in detecting GI lesions. “Specifically,” the analysis revealed that TXI significantly improves mean CD and visibility scores compared to WLE. “Furthermore,” the combined lesion detection rate, including adenomas, gastric neoplasms, and Barrett's esophagus, substantiated TXI's superiority over WLE.

TXI is a device‐based image enhancement technology designed to address the limitations of traditional WLE. The main issues encountered with WLE include non‐uniform illumination due to lumen curvature, challenges in color differentiation, and limited texture perception—all improved by TXI.^28^ Previously, non‐uniform illumination was addressed using histogram equalization and gamma correction image processing algorithms. These methods adjust grayscale values and brightness/contrast to enhance darker areas; however, individually processing pixels can produce significant differences from the original image, potentially misleading observers when assessing subtle mucosal changes. In contrast, TXI utilizes adaptive pixel enhancement relative to neighboring pixels and employs a modified retinex theory. TXI splits images into two layers—the base and detail layers—to enhance subtle tissue differences, and then applies corrections to preserve a natural, WLE‐like appearance while improving color visibility.^28^

A crucial aspect of assessing TXI's performance involves calculating CD, requiring an understanding of standardized color perception. To quantify CD, the CIE established the standardized CIE L*a*b* color space system.^29^ This space consists of three dimensions: (1) the L* axis, which plots a color point from black to white (white being the highest value); (2) the a* axis, which grades from red to green (red being the highest value); and (3) the b* axis, which grades from yellow to blue (yellow assigned the highest value). Visualizing this three‐dimensional space allows plotting color points, approximating human color perception. The distance between two points quantified as ΔE, represents measurable CD.^25^ All studies in our analysis utilized this standardized system, ensuring consistency and validating TXI's observed advantage in CD compared to WLE. Additionally, enhanced visibility scores demonstrate that TXI improves not only color contrast but also lesion morphology and texture recognition, crucial for accurate diagnosis and treatment planning.

An important comparison between TXI modes—TXI‐1 and TXI‐2—revealed that TXI‐1 significantly outperformed TXI‐2 in enhancing CD and lesion visibility. Unlike TXI‐2, which primarily improves brightness along with texture, TXI‐1 emphasizes color enhancement, making reddish mucosa appear redder and increasing contrast between red and white areas.^4^, ^36^ This improves lesion detection by clarifying small morphological changes. Additionally, our analysis showed lesion visibility was unaffected by endoscopist experience; identical detection trends were observed among experts and novices. This aligns with previous findings^4^, ^25^ indicating constant visibility enhancement across skill levels, facilitating accurate lesion identification even for trainees.

Our meta‐analysis also established TXI's superiority in specific GI tract regions, particularly the pharynx and esophagus. TXI notably improved mucosal contrast in the pharynx, facilitating lesion detection in this anatomically complex area.^7^, ^8^ Similarly, TXI‐1 significantly improved visibility in Barrett's esophagus compared to WLE and LCI. TXI‐1′s enhancement of color tone, brightness, and structural detail facilitated clearer visualization of palisade vessels and morphological changes characteristic of Barrett's mucosa. Although TXI‐2 provides texture enhancement useful for subtle morphological alterations, TXI‐1′s superior color tone enhancement makes it especially effective for identifying Barrett's esophagus.^30^

Previous IEE techniques like blue light imaging (BLI) and LCI have demonstrated some efficacy in early gastric cancer (EGC) detection,^31^, ^32^ but limitations such as insufficient brightness have prevented consistent outperformance of WLE. For example, Yoshida et al. found second‐generation NBI did not significantly improve EGC detection over WLE.^33^ Similarly, Japanese Guidelines state that the utility of enhanced endoscopy in EGC detection remains unclear.^34^ Our findings suggest that TXI's combination of texture and color enhancement with sufficient brightness offers improved lesion recognition over WLE and potentially other advanced technologies. Enhanced visualization of subtle mucosal changes and clearer color differentiation significantly contributed to TXI's effectiveness in detecting gastric neoplastic lesions.^35^

Detection and removal of colorectal neoplasia are primary objectives of colonoscopy, particularly in colorectal cancer screening. Our analysis demonstrated that TXI significantly improved colorectal lesion visibility scores and color differentiation over WLE and NBI, including both protruded and flat lesions. Importantly, these enhancements were achieved without extending withdrawal time or disrupting clinical workflows. Unlike NBI, whose effectiveness can be limited by luminal contents and reduced brightness, TXI maintains clarity even under suboptimal bowel preparation.^5^ This advantage significantly improves neoplasia detection rates in realistic clinical settings. TXI's ability to brighten darker areas and enhance subtle mucosal textures contributes markedly to its effectiveness in colorectal lesion detection.^30^

Considering broader implications, comparing TXI with other device‐based enhancement techniques and exploring its future prospects is critical. Technologies such as virtual chromoendoscopy methods (NBI and BLI for magnification endoscopy) and LCI for non‐magnified imaging similarly enhance anatomical details using specific wavelengths.^6^, ^32^ LCI, conceptually similar to TXI, employs a 410–450 nm wavelength to enhance white and red color details, mimicking the WLE image.^11^ While NBI, BLI, and LCI enhance lesion detection, limitations include reduced brightness, susceptibility to luminal content, and limited evidence from small‐scale studies. TXI distinguishes itself by employing standard white light combined with advanced processing algorithms that improve texture and color while preserving natural image appearance. This natural presentation not only enhances lesion detection but may facilitate clinical decision‐making. Conversely, Kadota et al. reported better gastric neoplasm detection using 3G‐NBI and WLE compared to TXI, despite our findings favoring TXI.^37^ Possible explanations include the learning curve associated with TXI, background mucosal enhancement obscuring lesions, or subtle CDs between inflamed and neoplastic mucosa. Additionally, optimal mode selection was not standardized, potentially influencing these outcomes.^37^ Nevertheless, our findings underscore TXI's clear clinical advantage in lesion detection compared to WLE, with a natural appearance aligned to human visual perception.

Despite these promising findings, several limitations must be acknowledged. First, the lack of direct comparative studies between TXI and other enhanced imaging modalities (NBI, BLI, and LCI) limits comprehensive positioning within the endoscopic technology spectrum. Second, the majority of included studies were conducted in Japan, potentially affecting generalizability to other populations and clinical settings. Additionally, although minimal heterogeneity was reported in analyses with higher numbers of studies, differences in study designs, endoscopic equipment, and patient populations in observational studies might contribute to variability. The limited number of high‐quality RCTs (only three included) and potential publication bias could further influence evidence quality. Future research should therefore emphasize direct comparative studies and larger multicenter trials.

## CONCLUSION

Our meta‐analysis provides compelling evidence that TXI significantly enhances GI lesion detection compared to WLE. By improving color differentiation and visibility scores across various GI regions, TXI effectively addresses major limitations of traditional and other advanced imaging modalities. Although additional studies comparing TXI directly with other modalities and examining different modes are warranted, current evidence strongly supports incorporating TXI into routine endoscopic practice to improve diagnostic outcomes.

## CONFLICT OF INTEREST STATEMENT

None.

## ETHICS STATEMENT

Approval of the Research Protocol by an Institutional Review Board: N/A

## PATIENT CONSENT STATEMENT

Not applicable to this study.

## CLINICAL TRIAL REGISTRATION

Not applicable to this study.

## Supporting information



Supplemental File 1.docx

Supplemental File 2.docx

## Data Availability

The datasets utilized and analyzed in this study are available from the corresponding author upon reasonable request.

## References

[1] Aalami AH , Abdeahad H , Mesgari M . Circulating miR‐21 as a potential biomarker in human digestive system carcinoma: A systematic review and diagnostic meta‐analysis. Biomarkers 2021; 26: 103–13. Available from: https://www.tandfonline.com/doi/full/10.1080/1354750X.2021.1875504 33434077 10.1080/1354750X.2021.1875504

[2] Choi HS , Hwang JH . Endoscopic resection of early luminal cancer. Gastrointest Endosc Clin N Am 2024; 34: 51–78. Available from: https://linkinghub.elsevier.com/retrieve/pii/S1052515723000661 37973231 10.1016/j.giec.2023.07.002

[3] Akarsu M , Akarsu C . Evaluation of new technologies in gastrointestinal endoscopy. J Soc Laparoendosc Surg 2018; 22 :e2017.00053. Available from: https://www.ncbi.nlm.nih.gov/pmc/articles/PMC5788542/ 10.4293/JSLS.2017.00053 PMC5788542 29398900

[4] Dobashi A , Ono S , Furuhashi H *et al*. Texture and Color enhancement imaging increases color changes and improves visibility for squamous cell carcinoma suspicious lesions in the pharynx and esophagus. Diagnostics 2021; 11: 1971. Available from: https://www.mdpi.com/2075‐4418/11/11/1971 34829318 10.3390/diagnostics11111971 PMC8622480

[5] Antonelli G , Bevivino G , Pecere S *et al*. Texture and color enhancement imaging versus high definition white‐light endoscopy for detection of colorectal neoplasia: A randomized trial. Endoscopy 2023; 55: 1072–80. Available from: https://pubmed.ncbi.nlm.nih.gov/37451283/ 37451283 10.1055/a-2129-7254

[6] Ikeda A , Takeda T , Ueyama H *et al*. Comparison of texture and color enhancement imaging with white light imaging in 52 patients with short‐segment Barrett's esophagus. Med Sci Monit 2023; 29: e940249. Available from: https://pubmed.ncbi.nlm.nih.gov/37309104/ 37309104 10.12659/MSM.940249 PMC10276532

[7] Sugimoto M , Kawai Y , Akimoto Y *et al*. Third‐generation high‐vision ultrathin endoscopy using texture and color enhancement imaging and narrow‐band imaging to evaluate Barrett's esophagus. Diagnostics 2022; 12: 3149. Available from: https://www.embase.com/search/results?subaction=viewrecord&id=L2020769266&from=export 10.3390/diagnostics12123149 PMC9777380 36553156

[8] Abe S , Makiguchi ME , Nonaka S , Suzuki H , Yoshinaga S , Saito Y . Emerging texture and color enhancement imaging in early gastric cancer. Gastroenterol Endosc 2022; 34: 714–20. Available from: https://www.embase.com/search/results?subaction=viewrecord&id=L2014341229&from=export 10.1111/den.14182 34716942

[9] Hiramatsu T , Nishizawa T , Kataoka Y , Yoshida S , Matsuno T , Mizutani H . Improved visibility of colorectal tumor by texture and color enhancement imaging with indigo carmine. World J Gastrointest Endosc 2023; 15: 690–8. Available from: https://www.wjgnet.com/1948‐5190/full/v15/i12/690.htm 38187913 10.4253/wjge.v15.i12.690 PMC10768041

[10] Toyoshima O , Nishizawa T , Yoshida S *et al*. Texture and color enhancement imaging in magnifying endoscopic evaluation of colorectal adenomas. World J Gastrointest Endosc 2022 Feb; 14 (2): 96–105. 35316981 10.4253/wjge.v14.i2.96 PMC8908327

[11] Sato T . TXI: Texture and color enhancement imaging for endoscopic image enhancement. J Healthc Eng 2021; 2021: 5518948. Available from: https://www.embase.com/search/results?subaction=viewrecord&id=L2011740672&from=export 33880168 10.1155/2021/5518948 PMC8049784

[12] Page MJ , McKenzie JE , Bossuyt PM . The PRISMA 2020 statement: An updated guideline for reporting systematic reviews. Bmj 2021; 372: n71. 33782057 10.1136/bmj.n71 PMC8005924

[13] Wells GA , Shea B , O'Connell D *et al*. The Newcastle‐Ottawa Scale (NOS) for assessing the quality of nonrandomised studies in meta‐analyses. 2000. Available from: https://www.ohri.ca/programs/clinical_epidemiology/oxford.asp

[14] GRADEpro GDT . GRADEpro Guideline Development Tool [Software]. McMaster University and Evidence Prime. 2023.

[15] Rücker G , Carpenter JR , Schwarzer G . Detecting and adjusting for small‐study effects in meta‐analysis. Biom J 2011; 53: 351–68. Available from: http://www.ncbi.nlm.nih.gov/pubmed/21374698 21374698 10.1002/bimj.201000151

[16] Abe S , Yamazaki T , Hisada IT *et al*. Visibility of early gastric cancer in texture and color enhancement imaging. DEN open 2022; 2: e46. 35310718 10.1002/deo2.46 PMC8828244

[17] Young E , Rajagopalan A , Tee D , Sathananthan D , Hoile S , Singh R . Texture and color enhancement imaging improves colonic adenoma detection: A multicenter randomized controlled trial. Gastroenterology 2024; 166: 338–40.e3. 37839498 10.1053/j.gastro.2023.10.008

[18] Okumura T , Hotta K , Imai K *et al*. Efficacy of texture and color enhancement imaging for the visibility and diagnostic accuracy of non‐polypoid colorectal lesions. DEN Open 2025; 5: 1–8. 10.1002/deo2.380 PMC11136699 38817687

[19] Kato T , Hikichi T , Nakamura J *et al*. Visibility of esophageal squamous cell carcinoma under iodine staining on texture and color enhancement imaging. DEN Open 2025; 5: e370. 38725874 10.1002/deo2.370 PMC11079435

[20] Kemmoto Y , Ozawa SI , Sueki R *et al*. Higher detectability of gastric cancer after Helicobacter pylori eradication in texture and color enhancement imaging mode 2 in screening endoscopy. DEN Open 2024; 4: e279. 37529380 10.1002/deo2.279 PMC10387742

[21] Sakamoto T , Ikematsu H , Tamai N *et al*. Detection of colorectal adenomas with texture and color enhancement imaging: Multicenter observational study. Dig Endosc 2023; 35: 529–37. Available from: https://onlinelibrary.wiley.com/doi/10.1111/den.14480 36398944 10.1111/den.14480 PMC12136281

[22] Yoshida N , Inoue K , Dohi O *et al*. Analysis of texture and color enhancement imaging for improving the visibility of non‐polypoid colorectal lesions. Dig Dis Sci 2022; 67: 5657–65. Available from: https://www.embase.com/search/results?subaction=viewrecord&id=L2015389140&from=export 35318554 10.1007/s10620-022-07460-5

[23] Koyama Y , Sugimoto M , Kawai T *et al*. Visibility of early gastric cancers by texture and color enhancement imaging using a high‐definition ultrathin transnasal endoscope. Sci Rep 2023; 13: 1994. Available from: https://www.embase.com/search/results?subaction=viewrecord&id=L640250448&from=export 36737509 10.1038/s41598-023-29284-7 PMC9898248

[24] Hiramatsu T , Nishizawa T , Kataoka Y *et al*. Improved visibility of colorectal tumor by texture and color enhancement imaging with indigo carmine. World J Gastrointest Endosc 2023; 15: 690–8. 38187913 10.4253/wjge.v15.i12.690 PMC10768041

[25] Nishizawa T , Toyoshima O , Yoshida S *et al*. TXI (Texture and color enhancement imaging) for serrated colorectal lesions. J Clin Med 2022; 11: 119. Available from: https://www.embase.com/search/results?subaction=viewrecord&id=L2015070109&from=export 10.3390/jcm11010119 PMC8745100 35011860

[26] Ishikawa T , Matsumura T , Okimoto K *et al*. Efficacy of texture and color enhancement imaging in visualizing gastric mucosal atrophy and gastric neoplasms. Sci Rep 2021; 11: 6910. Available from: https://www.embase.com/search/results?subaction=viewrecord&id=L634665692&from=export 33767278 10.1038/s41598-021-86296-x PMC7994634

[27] Futakuchi T , Dobashi A , Horiuchi H *et al*. Texture and color enhancement imaging improves the visibility of gastric neoplasms: Clinical trial with image catalogue assessment using conventional and newly developed endoscopes. BMC Gastroenterol 2023; 23: 389. 37957560 10.1186/s12876-023-03030-9 PMC10644425

[28] Wagner A , Zandanell S , Kiesslich T *et al*. Systematic review on optical diagnosis of early gastrointestinal neoplasia. J Clin Med 2021; 10: 2794. Available from: https://www.mdpi.com/2077‐0383/10/13/2794 34202001 10.3390/jcm10132794 PMC8269336

[29] Kuehni RG . Color‐tolerance data and the tentative CIE 1976 L*a*b* formula. J Opt Soc Am 1976; 66: 497. Available from: https://opg.optica.org/abstract.cfm?URI=josa‐66‐5‐497 932844 10.1364/josa.66.000497

[30] Tamai N , Horiuchi H , Matsui H *et al*. Visibility evaluation of colorectal lesion using texture and color enhancement imaging with video. DEN Open 2022; 2: e90. Available from: https://pubmed.ncbi.nlm.nih.gov/35310754/ 10.1002/deo2.90 PMC8828205 35310754

[31] Ono S , Kawada K , Dohi O *et al*. Linked color imaging focused on neoplasm detection in the upper gastrointestinal tract : A randomized trial. Ann Intern Med 2021; 174: 18–24. Available from: https://pubmed.ncbi.nlm.nih.gov/33076693/ 33076693 10.7326/M19-2561

[32] Yao K , Uedo N , Kamada T *et al*. Guidelines for endoscopic diagnosis of early gastric cancer. Dig Endosc 2020; 32: 663–98. Available from: https://pubmed.ncbi.nlm.nih.gov/32275342/ 32275342 10.1111/den.13684

[33] Paggi S , Radaelli F , Senore C *et al*. Linked‐color imaging versus white‐light colonoscopy in an organized colorectal cancer screening program. Gastrointest Endosc 2020; 92: 723–30. Available from: https://pubmed.ncbi.nlm.nih.gov/32502550/ 32502550 10.1016/j.gie.2020.05.044

[34] Yoshida N , Doyama H , Yano T *et al*. Early gastric cancer detection in high‐risk patients: A multicentre randomised controlled trial on the effect of second‐generation narrow band imaging. Gut 2021; 70: 67–75. Available from: https://pubmed.ncbi.nlm.nih.gov/32241898/ 32241898 10.1136/gutjnl-2019-319631 PMC7788198

[35] Dohi O , Yagi N , Naito Y *et al*. Blue laser imaging‐bright improves the real‐time detection rate of early gastric cancer: A randomized controlled study. Gastrointest Endosc 2019; 89: 47–57. Available from: https://pubmed.ncbi.nlm.nih.gov/30189197/ 30189197 10.1016/j.gie.2018.08.049

[36] Tamai N , Sumiyama K . Texture and color enhancement imaging (TXI). In: Sano Y , Chiu P , Singh R , Uedo N , Goda K , Katada C (eds). Atlas of Advanced Endoscopy, Singapore: Springer, 2024; 11–6.

[37] Kadota T , Abe S , Uedo N *et al*. Comparison of effective imaging modalities for detecting gastric neoplasms: A randomized three‐arm phase II trial. Off J Am Coll Gastroenterol 2024; 119: 2010–8. Available from: https://pubmed.ncbi.nlm.nih.gov/38752623/ 10.14309/ajg.0000000000002871 PMC11446510 38752623

