# Supplementary material for: Assessing the effectiveness of texture and color enhancement imaging versus white‐light endoscopy in detecting gastrointestinal lesions: A systematic review and meta‐analysis
Source: DEN Open. 2025 Apr 30;6(1):e70128. doi: 10.1002/deo2.70128 (PMC12044138; doi:10.1002/deo2.70128)
Supplement: Supplementary file 1 — Supplemental File 1.docx [file DEO2-6-e70128-s002.docx]

**Supplementary Figure 1**

**
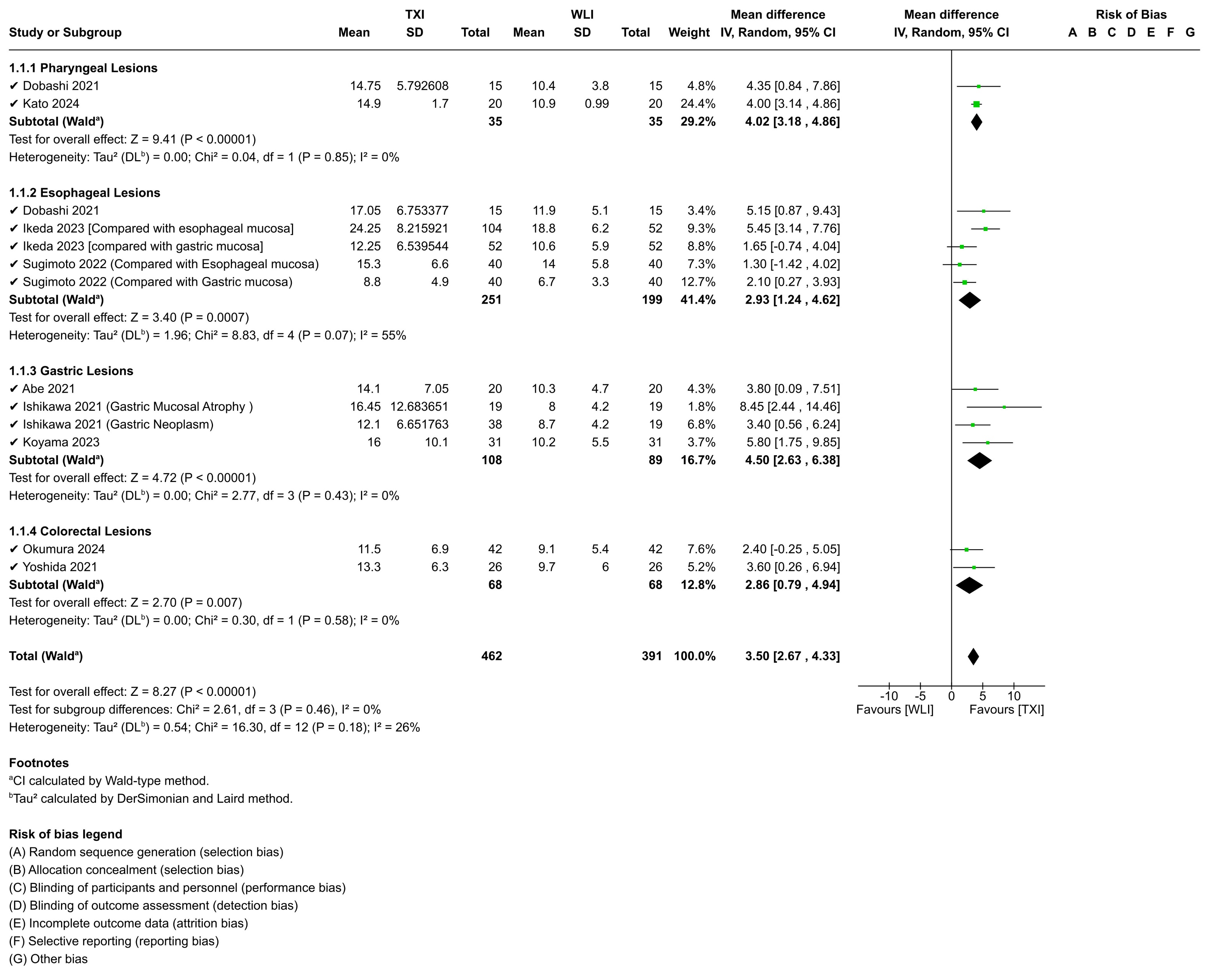
**

**Supplementary Figure 2**

**
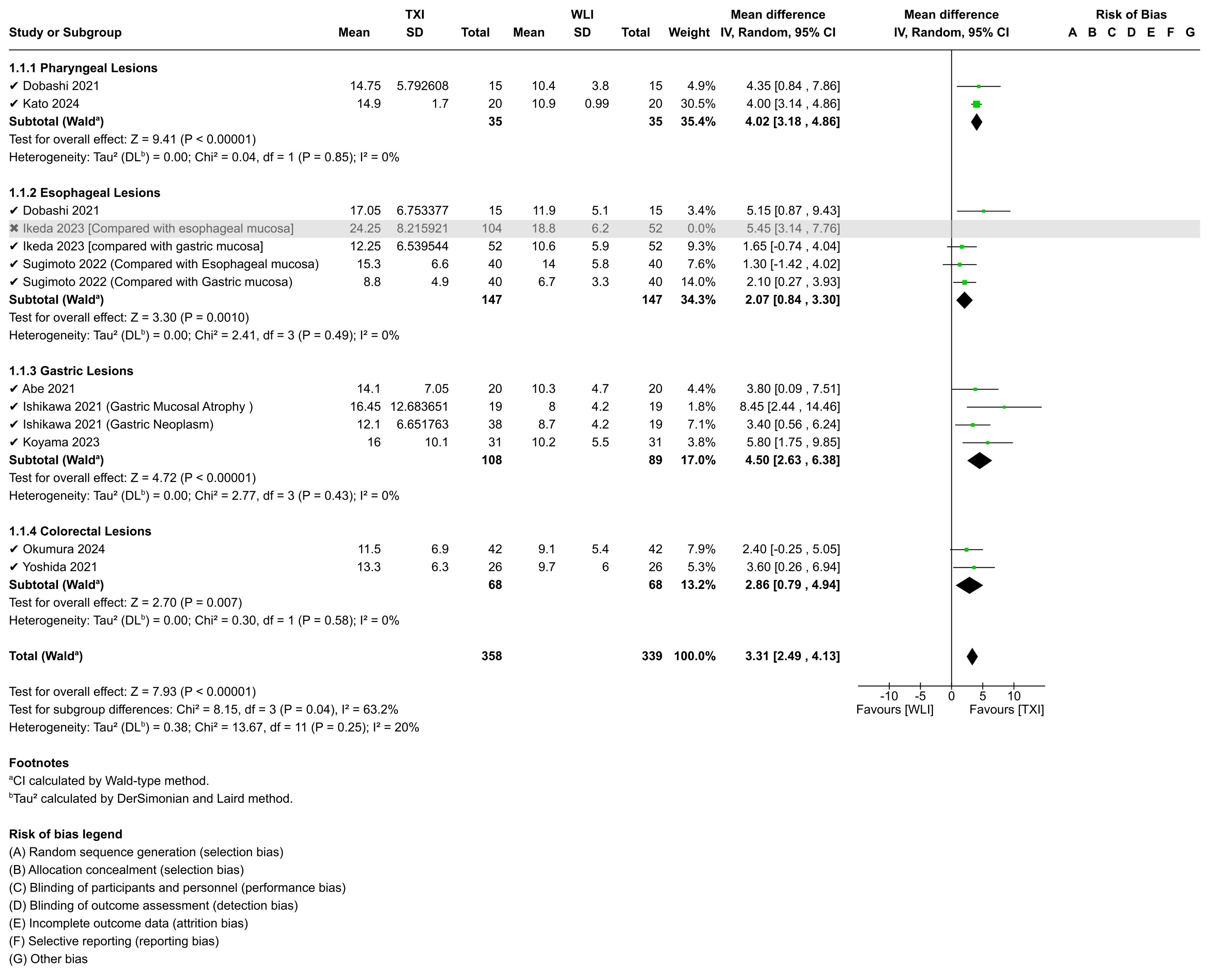
**

**Supplementary Figure 3**

**
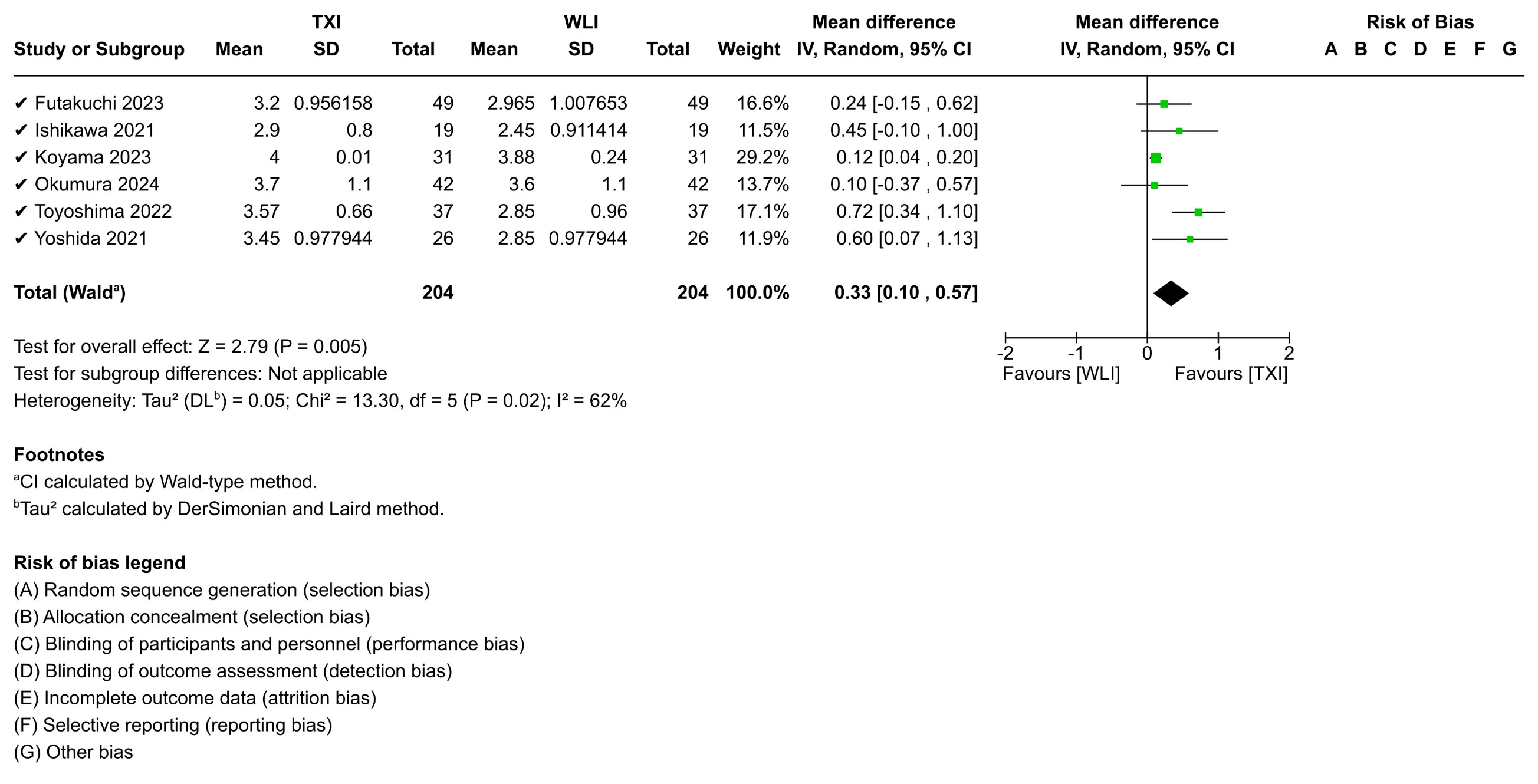
**

**Supplementary Figure 4**

**
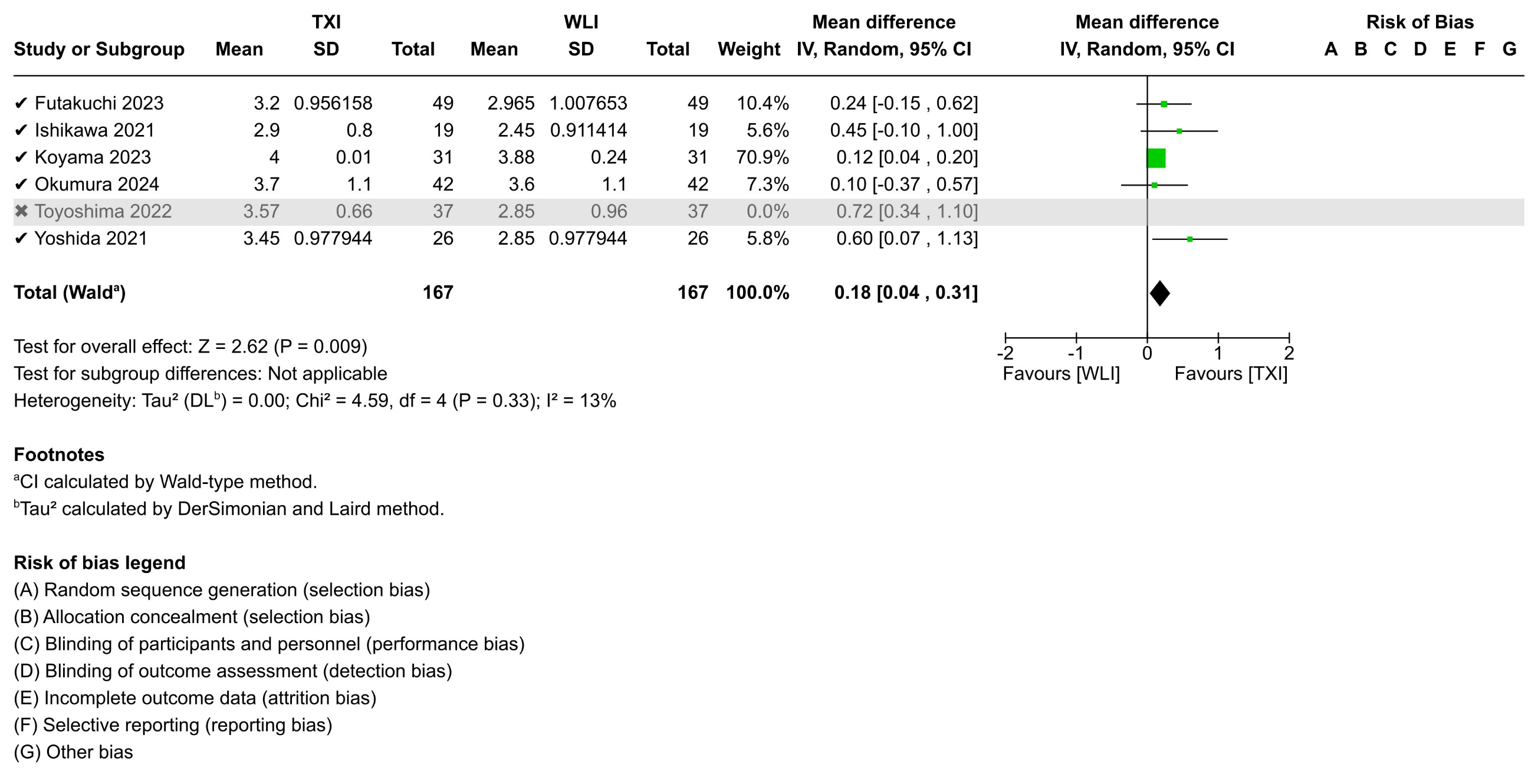
**

**Supplementary Figure 5**

**
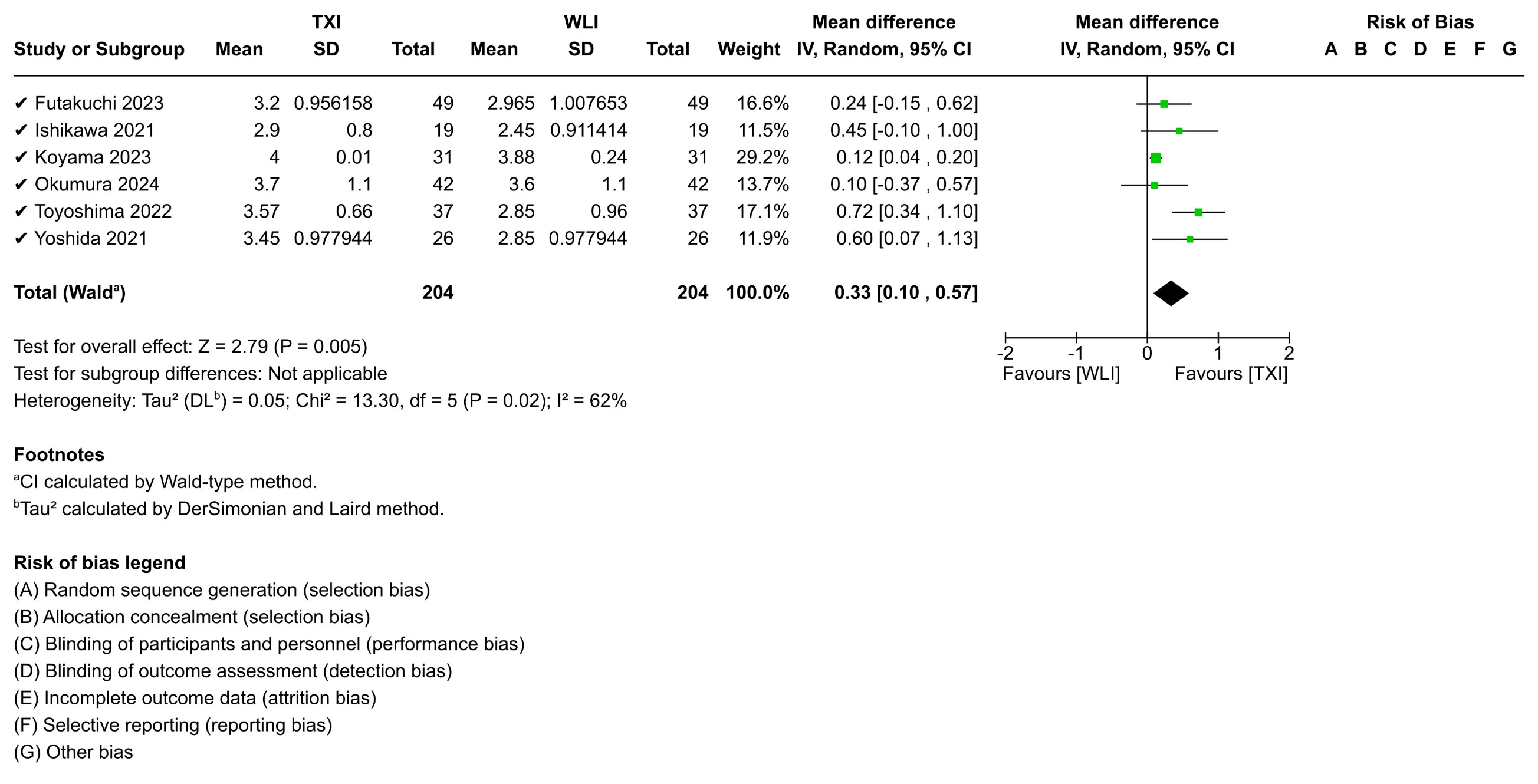
**

**Supplementary Figure 6**

**
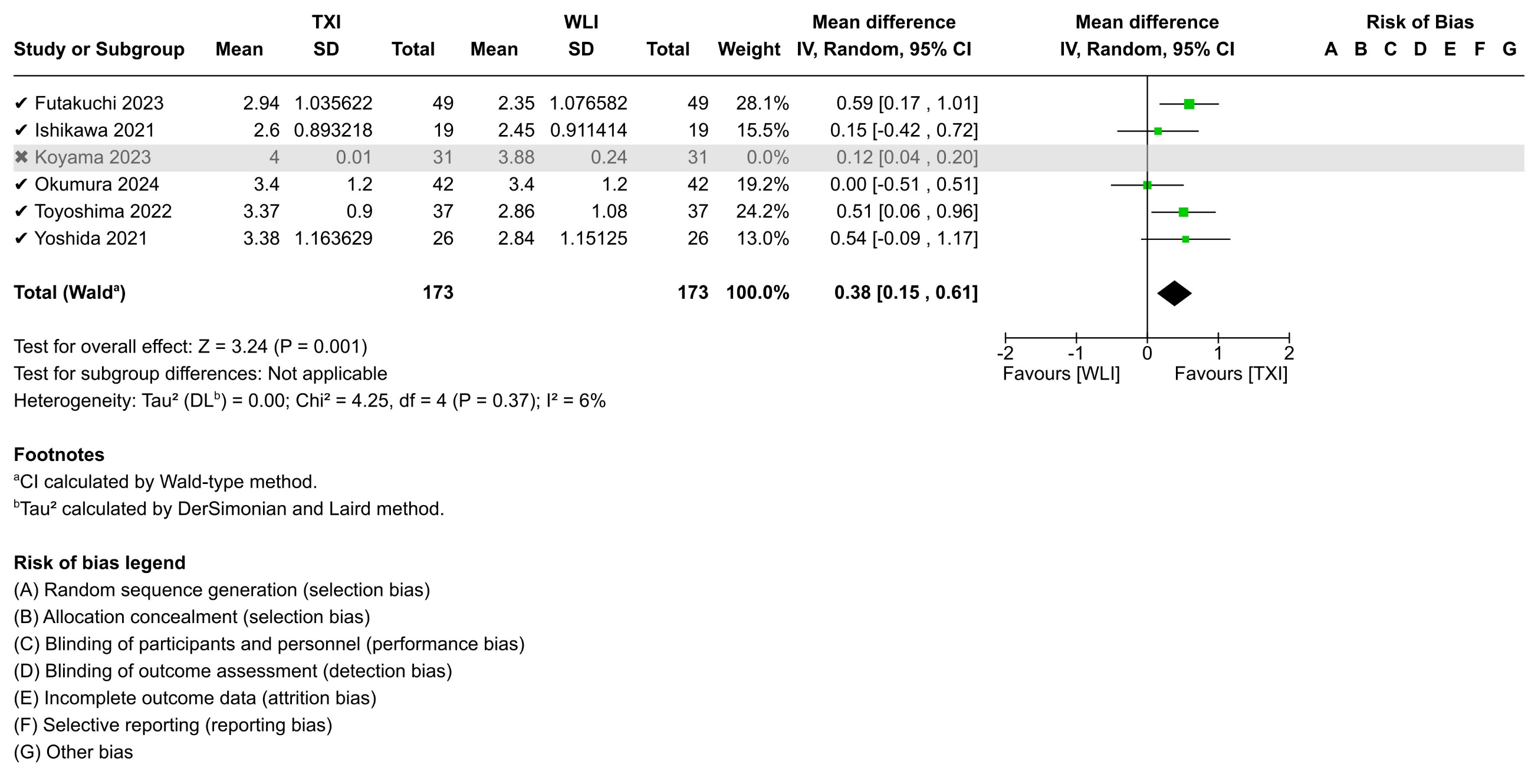
**

**Supplementary Figure 7**

**
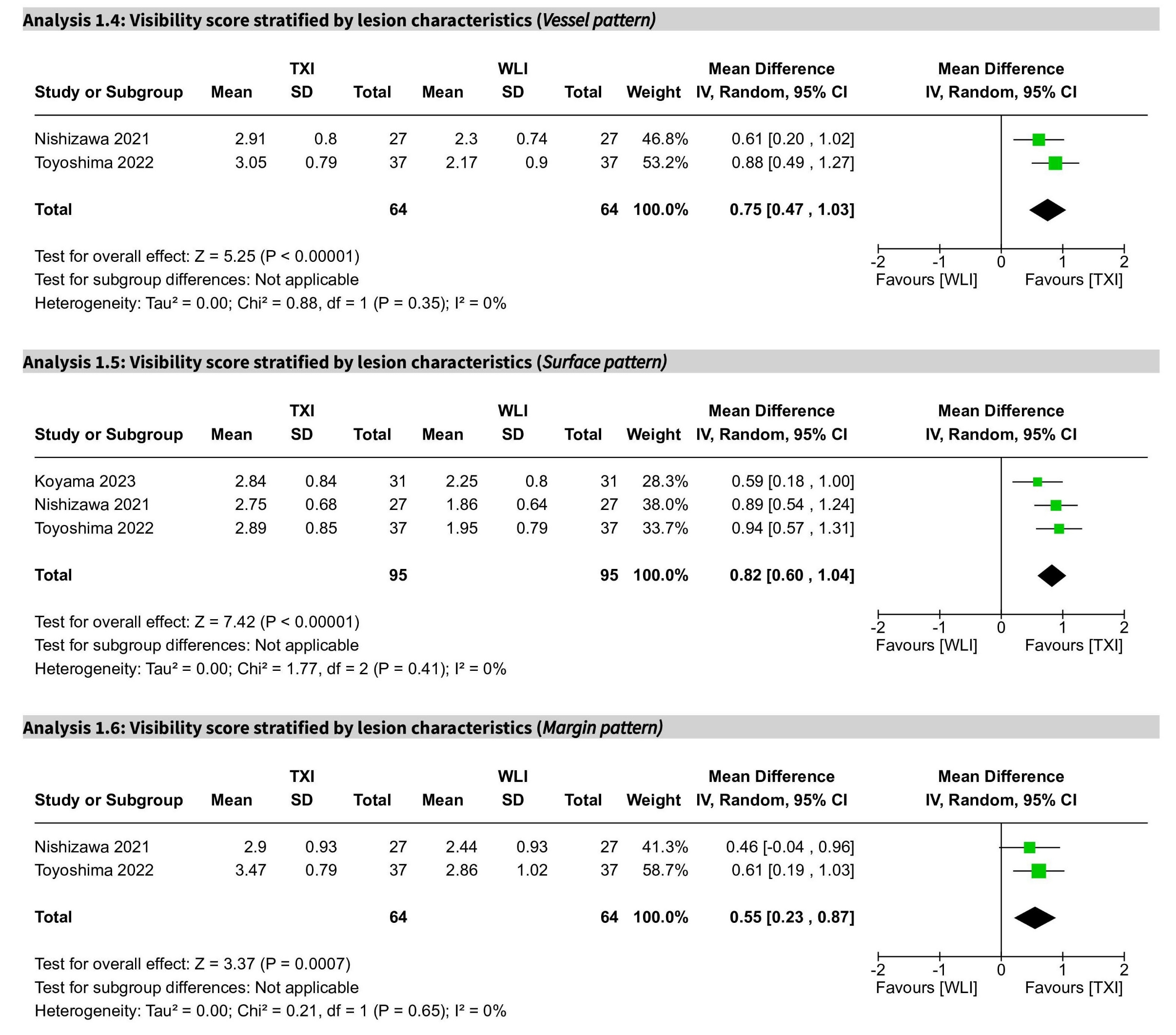
**

**Supplementary Figure 8**

**
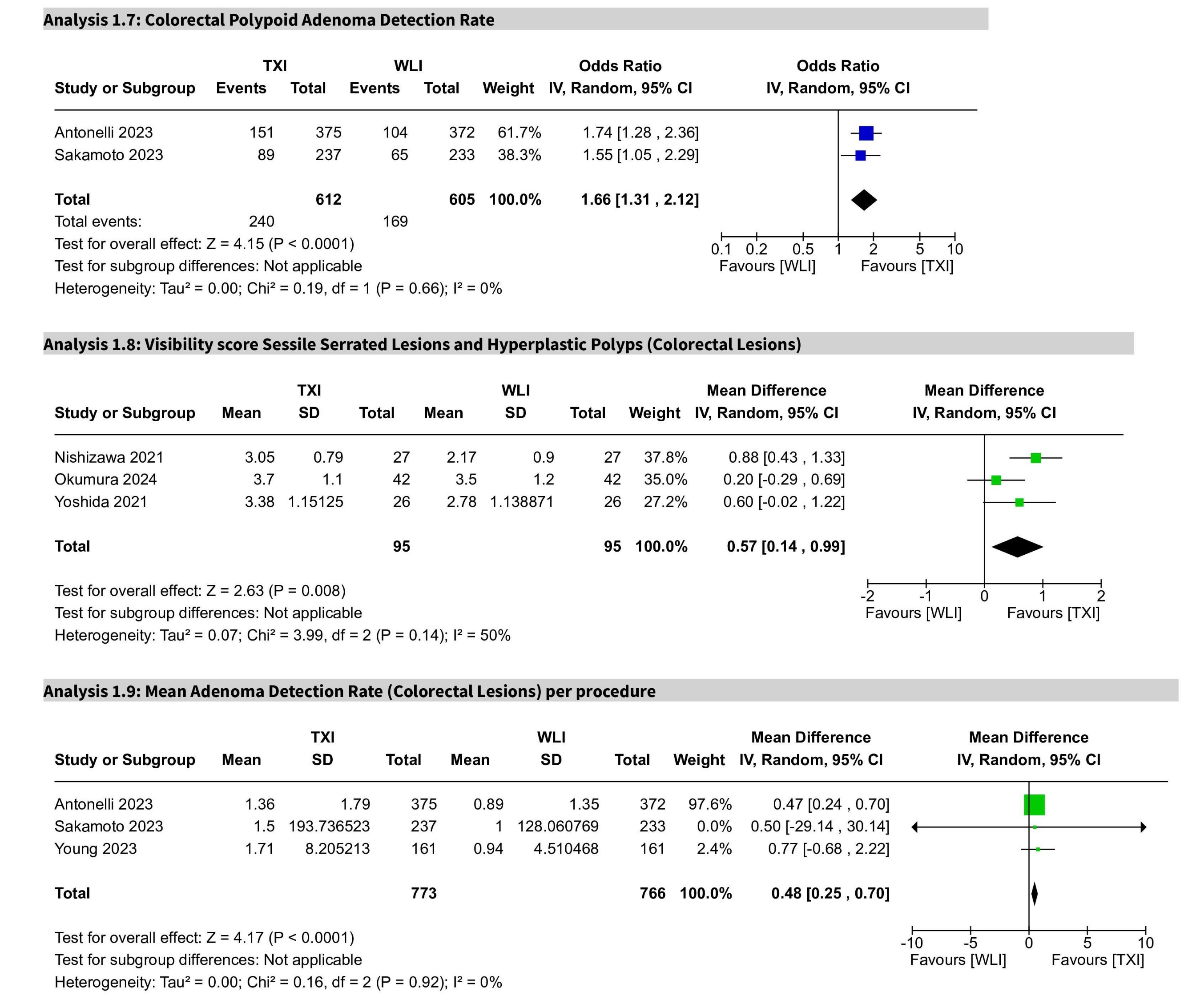
**

**Table S1. Detailed search strategy used for database searches**

| **Database** | **Search Terms** |
| --- | --- |
| PubMed | ("Textur*" AND "colo*" AND "enhanc*") OR ("texture*" AND "color enhanc*") OR "TXI" |
| Embase | #1 ('textur*' AND 'colo*' AND 'enhanc*') OR ('texture*' AND 'color enhanc*') OR 'TXI' |
|  | #2 ('textur*'/exp AND 'colo*'/exp AND 'enhanc*'/exp) OR ('texture*'/exp AND 'color enhanc*'/exp) OR 'TXI' |
|  | #3 #1 OR #2 |
| Cochrane | #1 ("Textur*" AND "colo*" AND "enhanc*") OR ("texture*" AND "color enhanc*") OR "TXI" |
|  | #2 ("Textur*") AND ("colo*") AND ("enhanc*") |
|  | #3 #1 OR #2 |
| Web of Science | ("Textur*" AND "colo*" AND "enhanc*") OR ("texture*" AND "color enhanc*") OR "TXI" |

**Table S2. GRADE assessment of the certainty of evidence**

| **Certainty assessment** | | | | | | | **№ of patients** | | **Effect** | | **Certainty** | **Importance** |
| --- | --- | --- | --- | --- | --- | --- | --- | --- | --- | --- | --- | --- |
| **№ of studies** | **Study design** | **Risk of bias** | **Inconsistency** | **Indirectness** | **Imprecision** | **Other considerations** | **New Analysis group** | **[placebo]** | **Relative (95% CI)** | **Absolute (95% CI)** |  |  |
| **Color difference between lesion and surrounding mucosa (mean + SD) - total** | | | | | | | | | | | | |
| 11 | non-randomised studies | not serious | not serious | not serious | not serious | **very strong association** | 358 | 339 | - | MD **3.31 higher** (2.49 higher to 4.13 higher) | ⨁⨁⨁⨁ High |  |
| **Visibility score of the lesion (mean ± SD) - total** | | | | | | | | | | | | |
| 7 | non-randomised studies | not serious | not serious | not serious | not serious | none | 302 | 302 | - | MD **0.5 higher** (0.36 higher to 0.64 higher) | ⨁⨁⨁⨁ High |  |
| **Gatrointestinal Lesion Detection rate - total** | | | | | | | | | | | | |
| 5 | non-randomised studies | not serious | not serious | not serious | not serious | none | 503/3510 (14.3%) | 398/11551 (3.4%) | **OR 1.84** (1.52 to 2.22) | **27 more per 1,000** (from 17 more to 39 more) | ⨁⨁⨁◯ Moderate |  |
| **Visibility score stratified by lesion characteristics (Vessel pattern)** | | | | | | | | | | | | |
| 2 | non-randomised studies | not serious | serious | not serious | not serious | none | 64 | 64 | - | MD **0.75 higher** (0.47 higher to 1.03 higher) | ⨁⨁◯◯ Low |  |
| **Visibility score stratified by lesion characteristics (Surface pattern)** | | | | | | | | | | | | |
| 3 | non-randomised studies | not serious | not serious | not serious | not serious | none | 95 | 95 | - | MD **0.82 higher** (0.6 higher to 1.04 higher) | ⨁⨁⨁◯ Moderate |  |
| **Visibility score stratified by lesion characteristics (Margin pattern)** | | | | | | | | | | | | |
| 2 | non-randomised studies | not serious | serious | not serious | not serious | none | 64 | 64 | - | MD **0.55 higher** (0.23 higher to 0.87 higher) | ⨁⨁◯◯ Low |  |
| **Colorectal Polypoid Adenoma Detection Rate** | | | | | | | | | | | | |
| 2 | non-randomised studies | not serious | serious | not serious | not serious | none | 240/612 (39.2%) | 169/605 (27.9%) | **OR 1.66** (1.31 to 2.12) | **112 more per 1,000** (from 57 more to 172 more) | ⨁⨁◯◯ Low |  |
| **Visibility score Sessile Serrated Lesions and Hyperplastic Polyps (Colorectal Lesions)** | | | | | | | | | | | | |
| 3 | non-randomised studies | not serious | serious | not serious | not serious | none | 95 | 95 | - | MD **0.57 higher** (0.14 higher to 0.99 higher) | ⨁⨁⨁◯ Moderate |  |
| **Mean Adenoma Detection Rate (Colorectal Lesions) per procedure** | | | | | | | | | | | | |
| 3 | non-randomised studies | not serious | not serious | not serious | not serious | none | 773 | 766 | - | MD **0.48 higher** (0.25 higher to 0.7 higher) | ⨁⨁⨁◯ Moderate |  |
| **Color difference between lesion and surrounding mucosa (mean + SD)** | | | | | | | | | | | | |
| **5** | non-randomised studies | not serious | not serious | not serious | not serious | **very strong association** | **140** | **140** | **-** | **MD 4.04 higher (2.24 higher to 5.85 higher)** | ⨁⨁⨁⨁ High |  |
| **Visibility score of the lesion (mean ± SD)** | | | | | | | | | | | | |
| **2** | non-randomised studies | not serious | serious | not serious | not serious | none | **79** | **79** | **-** | **MD 0.18 higher (0.02 higher to 0.35 higher)** | ⨁⨁◯◯ Low |  |
| **Visibility score of the lesion** | | | | | | | | | | | | |
| **6** | non-randomised studies | not serious | serious | not serious | not serious | none | **204** | **204** | **-** | **MD 0.05 higher (0.05 lower to 0.14 higher)** | ⨁⨁⨁⨁ High |  |

**CI:** confidence interval; **MD:** mean difference; **OR:** odds ratio
