# Supplementary material for: Assessing the effectiveness of texture and color enhancement imaging versus white‐light endoscopy in detecting gastrointestinal lesions: A systematic review and meta‐analysis
Source: DEN Open. 2025 Apr 30;6(1):e70128. doi: 10.1002/deo2.70128 (PMC12044138; doi:10.1002/deo2.70128)
Supplement: Supplementary file 2 — Supplemental File 2.docx [file DEO2-6-e70128-s001.docx]

***Color Difference Between Lesion and Surrounding Mucosa***

***
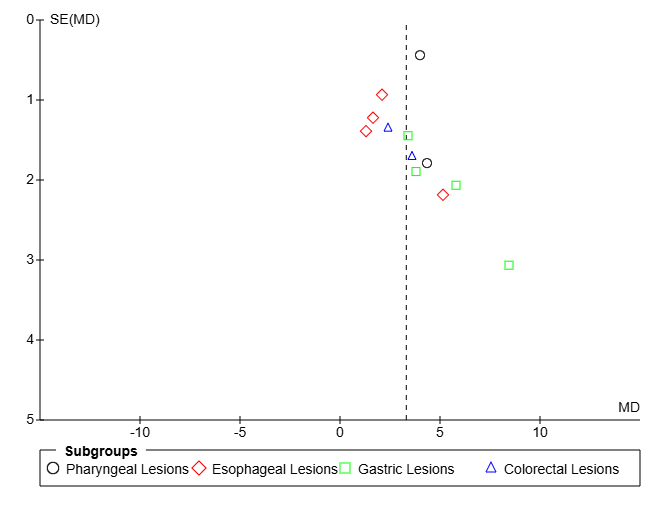
***

***Visibility Score of the Lesion***

***
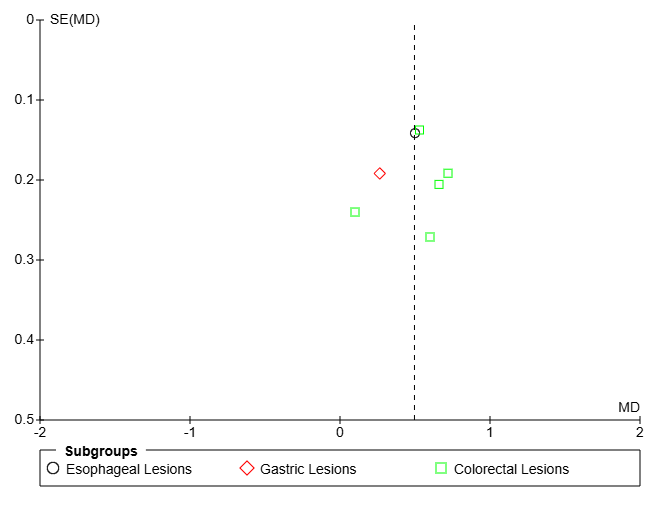
***

***Gastrointestinal Lesion Detection Rate***

***
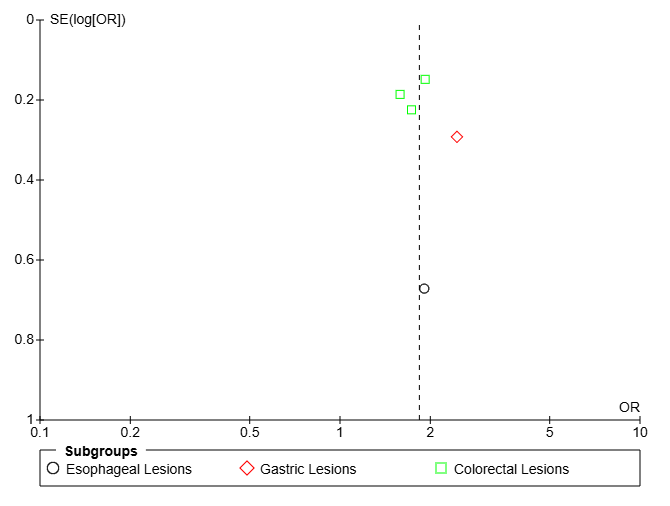
***

***TXI-Mode 1 vs TXI-Mode 2:***

***
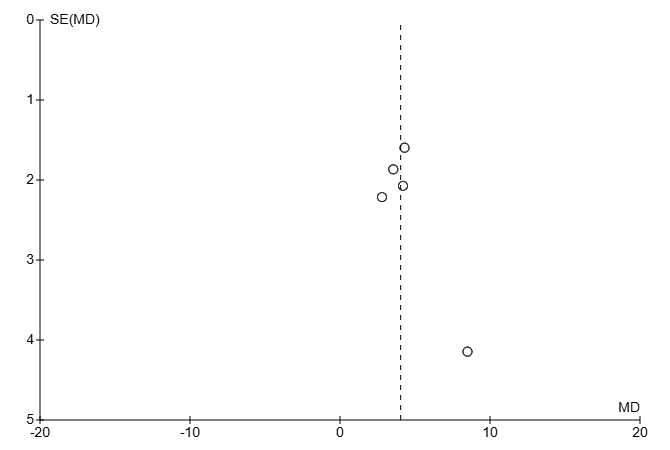
***

***Experts’ vs Trainees:***

***
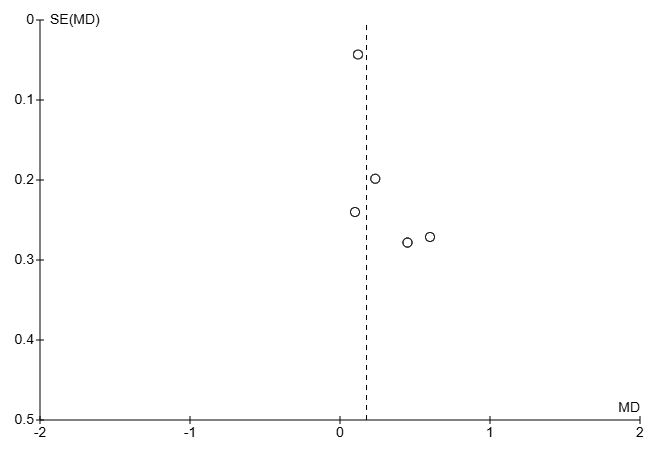
***

***
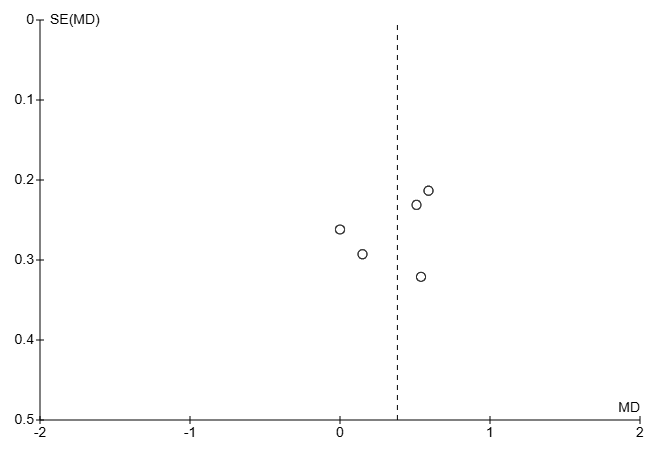
***

***
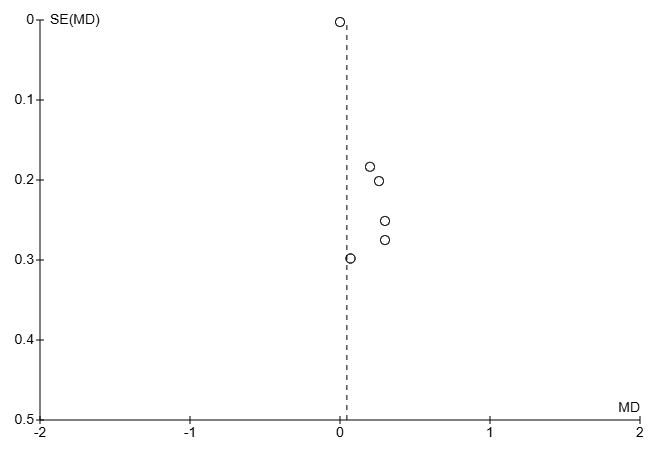
***
